# The impact of COVID-19 school disruptions on children’s learning

**DOI:** 10.3389/feduc.2024.1295910

**Published:** 2024-03-27

**Authors:** Courtney K. Blackwell, Maxwell Mansolf, Sean C. L. Deoni, Jody M. Ganiban, Leslie D. Leve, Amy E. Margolis, Monica McGrath, Sara S. Nozadi, T. Michael O’Shea, Phillip Sherlock, Qi Zhao, Kaja Z. LeWinn

**Affiliations:** 1 Department of Medical Social Sciences, Northwestern University Feinberg School of Medicine, Chicago, IL, United States; 2 Bill & Melinda Gates Foundation, Seattle, WA, United States; 3 Department of Psychological and Brain Sciences, George Washington University, Washington, DC, United States; 4 Counseling Psychology and Human Services, University of Oregon College of Education, Eugene, OR, United States; 5 Irving Medical Center, New York State Psychiatric Institute, Columbia University, New York, NY, United States; 6 Johns Hopkins Bloomberg School of Public Health, Baltimore, MD, United States; 7 Community Environmental Health Program, College of Pharmacy, University of New Mexico Health Sciences Center, Albuquerque, NM, United States; 8 University of North Carolina School of Medicine, Chapel Hill, NC, United States; 9 Anita Zucker Center for Excellence in Early Childhood Studies, University of Florida, Gainesville, FL, United States; 10 Department of Preventative Medicine, University of Tennessee Health Science Center, Memphis, TN, United States; 11 Department of Psychiatry, University of California, San Francisco, San Francisco, CA, United States

**Keywords:** COVID-19, pandemic, academic achievement, school closure, learning, children

## Abstract

**Introduction:**

National health policies to stop the spread of the COVID-19 virus in the US resulted in widespread school closures and disrupted learning in Spring 2020.

**Methods:**

This study draws on unique individual-level data from *n* = 282 5–12 year olds enrolled in the NIH Environmental influences on Child Health Outcomes (ECHO) Research Program to investigate associations between caregiver-reported duration of Spring 2020 learning disruptions and academic achievement.

**Results:**

Linear regression analyses estimated that children who experienced more than 4 weeks of instruction disruptions in Spring 2020 scored 4.5 points [95% CI: −8.77, −0.22] lower on age-normed math assessments compared to peers who had four or fewer weeks of disruption, adjusting for sociodemographic variables, pre-pandemic vocabulary, and COVID-19 family hardships and stress. No differences were found for reading. Children whose caregivers had higher levels of pandemic-related traumatic stress and lower educational attainment also had lower math scores, adjusting for all other covariates.

**Discussion:**

Results suggest educators and schools focus additional attention on supporting math instruction for children who experienced extended learning disruptions.

## 1 Introduction

COVID-19-related school closures affected more than 90% of children worldwide (UNESCO, 2021), including over 55 million in the US (Peele et al., 2021). With the unexpected nature of the pandemic and subsequent closures, schools varied in how and when they made the transition from in-person to remote instruction. For more than a quarter of US school districts, it took over 3 weeks to begin remote learning (Malkus, 2020), and 1 month into school closures, only 37% districts were providing a formal remote learning curriculum with instruction (Lake and Dusseault, 2020). Understanding whether and to what extent differences in how long schools did not provide instruction of any kind (remote or in-person) impacted student achievement is critical for informing pandemic recovery efforts.

### 1.1 Potential impact of COVID-19 learning disruptions on academic achievement

Emerging work on COVID-19 learning disruptions primarily drawing on group-level data points to average group-level negative pandemic-related effects on achievement of 0.08–0.18 standard deviations, with generally worse outcomes in math compared to reading; disparities by socioeconomic status, race, ethnicity, and grade level have also been noted (for reviews, see Hammerstein et al., 2021; Zierer, 2021; Cohodes et al., 2022; König and Frey, 2022; Betthäuser et al., 2023). For example, Molnár and Hermann (2023) found Hungarian children in schools with higher proportions of low-SES students experienced little or no academic progress during pandemic-related school disruptions, replicating work by Engzell et al. (2021) who showed Dutch students, particularly those from lower SES households, made no gains on standard exams following COVID-19 school disruptions. In the US, most studies have been limited to school-level poverty indicators, with students in high-poverty schools more negatively impacted compared to those in low-poverty schools (e.g., Goldhaber et al., 2022a,b; Kuhfeld et al., 2022a, 2023). Findings by race and ethnicity suggest US Black and Hispanic students experienced greater declines in achievement compared to White and non-Hispanic students (e.g., Goldhaber et al., 2022a,b; Kuhfeld et al., 2022a,b, 2023). Several international studies also suggest younger students were harder hit by the pandemic (e.g., Tomasik et al., 2021; Molnár and Hermann, 2023). In the US, large-scale studies primarily focus on grades 3–8 and report 3–5th graders had larger declines in achievement compared to 6–8th graders (e.g., Goldhaber et al., 2022a,b; Kuhfeld et al., 2022a, 2023).

Despite concordance of emerging evidence, existing work is limited in its generalizability and specificity. Most studies have been conducted outside the US and with more homogenous samples, drawing on group-level historical comparisons of achievement trends without adjustment for important individual-level confounders that may help explain variation in outcomes and identify alternative intervention targets beyond remedial education (Hammerstein et al., 2021; König and Frey, 2022; Betthäuser et al., 2023). Even the most rigorous US studies that include individual-level achievement data are limited because they all use the same dataset (MAP Growth assessment data) and lack important individual-level sociodemographic and contextual factors (e.g., family stressors). This results in conclusions that attribute any change over time to the pandemic or, in some cases, school-level characteristics, even if such differences may be due to more proximal individual-level differences. For example, Kuhfeld et al. (2022b) evaluated within-child changes in achievement from Fall 2019 to Spring 2021 for 5 million 3rd–8th graders in US public schools and differences by school-and district-level racial composition but could not evaluate whether changes in achievement differed by individual-level SES or adjust for any other individual-level covariates. Goldhaber et al. (2022a) extended this work to evaluate individual-level achievement changes through Fall 2021 and include individual-level data on race but were similarly limited by the lack of individual-level SES. In a follow-up study, the authors acknowledge lacking the necessary data to evaluate additional factors, such as family stressors outside of the school environment, which might explain the significant relationship between school closures and achievement (Goldhaber et al., 2022b). The most recent meta-analysis of 42 international studies reported most extant work was at serious or critical risk of bias due to confounding (Betthäuser et al., 2023). Much of the variance in achievement outcomes remains unaccounted for and attributed to pandemic-related differences when other factors such as SES and family stress may be driving such differences. Thus, despite the large sample sizes and robust methodological frameworks utilized in a handful of prior studies, there remains a dearth of research that incorporates critical individual-level factors.

To overcome these limitations, the current study leverages individual-level data from a diverse sample of 5–12-year-old US children to investigate associations between the duration of Spring 2020 COVID-19 learning disruptions and student achievement. We hypothesized longer learning disruptions in Spring 2020 where students received no formal instruction of any kind, whether in-person or remote, would be associated with worse math and reading achievement compared to shorter learning disruptions. Additionally, we conducted exploratory analyses of effect modification by child and family characteristics to understand whether learning disruptions differentially impacted achievement outcomes based on such factors.

## 2 Materials and methods

Data came from the Environmental influences on Child Health Outcomes (ECHO) research program. An NIH-funded research consortia comprised of existing longitudinal observational pediatric cohorts, ECHO aims to evaluate a range of early environmental exposures (e.g., biological, chemical, social) on five child outcomes: pre/peri/postnatal (e.g., preterm birth), airways (e.g., asthma), obesity, neurodevelopment, and positive health (e.g., well-being; LeWinn et al., 2022; Knapp et al., 2023).

### 2.1 Participants

Two ECHO cohorts contributed data as part of a COVID-19 supplemental award. The Conditions Affecting Neurocognitive Development and Learning in Early childhood (CANDLE) cohort enrolled pregnant individuals at four hospitals in Memphis, TN between 2006 and 2011. Participants in the CANDLE study reflect the demographics of Shelby County, TN, with the majority of caregivers identifying as Black (63%) and having lower income (64% < $50,000/year; Sontag-Padilla et al., 2015; LeWinn et al., 2020). The Brown University Assessment of Myelination and Behavioral development Across Maturation (BAMBAM) cohort was designed as an accelerated longitudinal study with a community-based sample of healthy children from the Providence, RI area. Recruited began in 2010, with approximately half enrolled between 2 and 8 months of age and half between 2 and 4 years of age (Deoni et al., 2012). The subsample of participants included in this study (CANDLE: *n* = 151; BAMBAM: *n* = 131) were those with data on the primary exposure (COVID-19 instruction disruption) and outcome (AAB). Primary caregivers consented into ECHO and provided consent for their children to participate. Local Institutional Review Boards (IRB) and the ECHO central IRB approved all data collection procedures.

### 2.2 Measures

#### 2.2.1 Academic achievement

**FIGURE 1:**
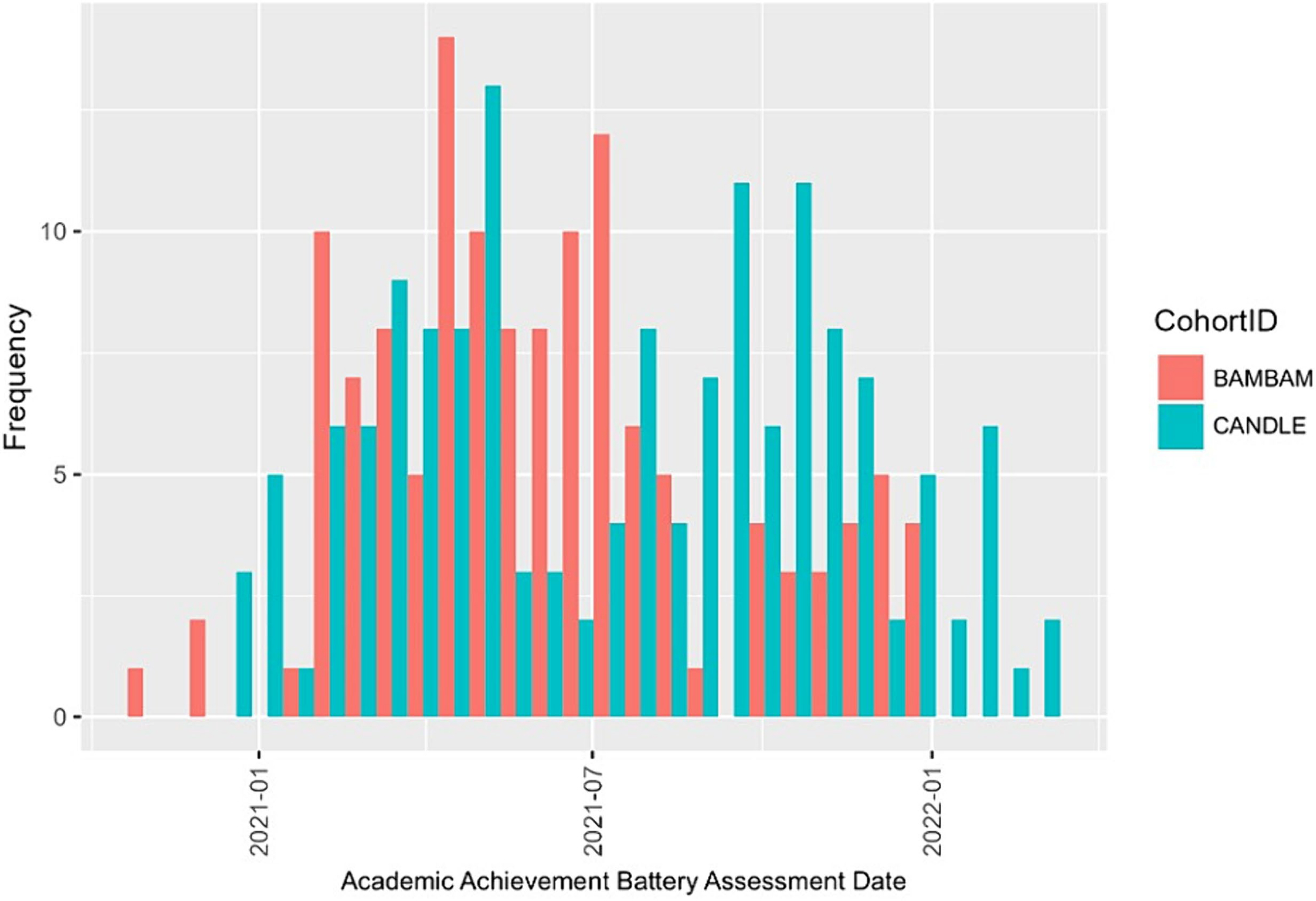
Academic achievement battery assessment date distribution by Cohort. Distribution of academic achievement battery assessment dates by ECHO Cohort. The first assessment occurred in October 2020 and the last assessment occurred in March 2022.

Cohorts administered two subtests from the Academic Achievement Battery (AAB; Messer, 2014) to children in-person. The Letter/Word Reading subtest evaluates components of basic reading skills, including letter identification and word pronunciation (Cronbach’s *α* = 0.94), and the Mathematical Calculation subtest assesses basic math skills by asking participants to provide oral and written responses to math problems and calculations (Cronbach’s *α* = 0.92). The AAB is age-and grade-normed and aligns with national academic standards (e.g., Common Core). For this study, age-based norms were used. We restricted data to AAB scores obtained after the end of the 2019/2020 school year (i.e., post-Spring 2020), defined as on or after May 23rd, 2020, for the CANDLE cohort based on the Shelby County School District calendar, the county in which CANDLE participants live and go to school; and on or after June 26th, 2020, for the BAMBAM cohort based on the Rhode Island Public School calendar, which applies to all public schools in the state. To account for the *n* = 19 children from BAMBAM with more than one AAB administration during the study period, we selected scores from the assessment closest to June 26th, 2020. No repeated AAB administrations were present for CANDLE. This resulted in AAB assessments occurring between October 2020 and March 2022 (Figure 1). We also derived a variable describing the *number of months from end of Spring 2020 to AAB*, reflecting the time between when schools ended their school year (as described above) to when children completed the AAB.

#### 2.2.2 Spring 2020 instruction disruption

Caregivers reported the number of weeks their child’s school was closed to instruction of any kind—in-person or remote—in Spring 2020. This represented the time between when the child’s school building physically closed and when learning resumed in-person or remotely in Spring 2020. Response options included: *no break between when the child’s school closed and when instruction resumed*, *less than 1 week*, *1 week*, *2 weeks*, *3 weeks*, *4 weeks*, and *more than 4 weeks*. We derived a dichotomous variable where >4 weeks closed = 1 (“extended disruption” group) and 4 weeks = 0 (“non-extended disruption” group). We selected this threshold because anything less is similar to the cumulative standard breaks students experience throughout the school year, such as winter and spring vacations. Additionally, missing school for >4 weeks represents missing 11% or more of the standard 180 instructional days per school year required in most states (including Tennessee and Rhode Island), which is just above the general 10% threshold used for chronic absenteeism in the US (Lara et al., 2018).

#### 2.2.3 Pre-pandemic academic functioning

**FIGURE 2:**
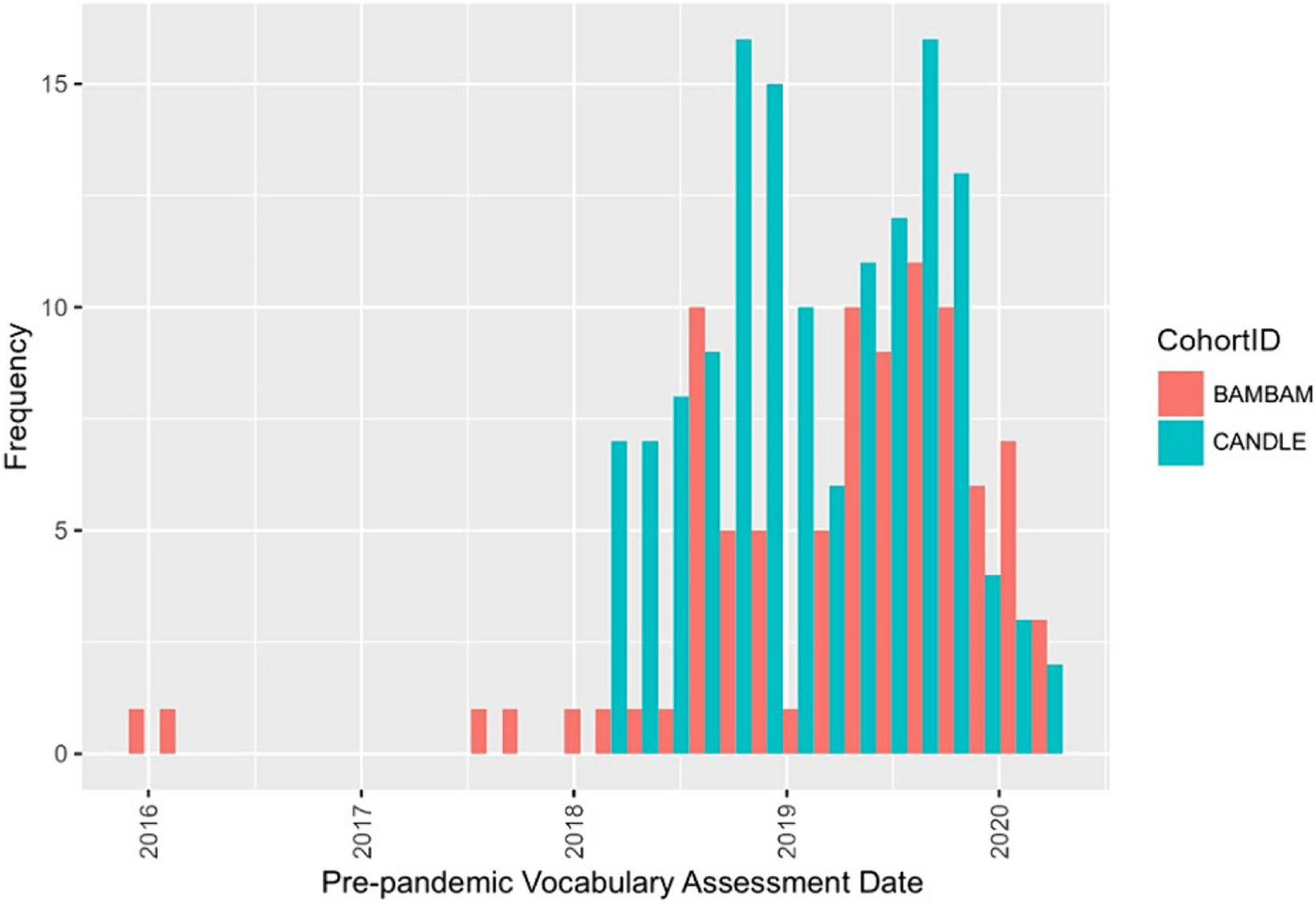
Pre-pandemic vocabulary assessment date distribution by Cohort. Distribution of harmonized pre-pandemic vocabulary assessment date by ECHO cohort. Dates ranged from December 2015 to March 2020.

In the absence of pre-pandemic AAB data, we controlled for pre-pandemic vocabulary scores, as vocabulary is a predictor of both reading and math achievement (Bleses et al., 2016). For most children (*n* = 206), vocabulary was measured using the NIH Toolbox Picture Vocabulary subtest (Gershon et al., 2013), and another 23 children had data on the Wechsler Intelligence Scale for Children, 5th Edition (WISC-5) Vocabulary subtest (Wechsler, 2014). Both instruments are norm-referenced to the general US population, use image-based stimuli, and correlated *r* = 0.72 for children in this sample with data on both measures (*n* = 42), reflecting excellent convergent validity (Weintraub et al., 2013). We therefore combined these data to maximize sample size. We linearly transformed vocabulary scores from T-scores to standard scores (*standard* = [*T*−50]/10*15 + 100) used by the AAB. We restricted vocabulary data to those completed on or before March 12th, 2020, reflecting the last day before the declaration of a national US emergency when schools in Shelby County and Rhode Island closed. We selected the assessment closest to March 12th, 2020 if children had more than one, resulting in assessments occurring between December 1, 2015 and March 7, 2020 (Figure 2). To account for differences in the time between the vocabulary assessment and AAB administration, we derived and controlled for a variable quantifying the *number of months between assessments.*

#### 2.2.4 Pandemic-related experiences

Caregivers reported whether they had difficulty accessing (1) food and (2) personal care products or household supplies; and whether they and/or their partner (3) became unemployed, (4) increased or decreased work hours, and (5) had a job that placed them at high risk for contracting coronavirus (Thomason et al., 2020). Items were summed to create a total *COVID-19 family hardships* score. Caregivers also completed the *Pandemic-related Traumatic Stress Scale* (*PTSS*; Cronbach’s *α* = 0.84; Blackwell et al., 2023), which is a 9-item scale aligned to Diagnostic and Statistical Manual of Mental Disorders, 5th Edition (DSM-5) Acute Stress Disorder criteria (American Psychiatric Association, 2022). Items were framed as, “Since becoming aware of the COVID-19 outbreak, how often have you..” using a 5-point Likert response scale anchored by *not at all* and *very often*. Average total scores were computed.

#### 2.2.5 Additional covariates

Variables included: *child individualized education plan (IEP)* described whether the child had an IEP (Yes = 1); *child age in years* (continuous) at AAB administration; *child sex* (reference = female); *caregiver educational attainment*, a dichotomous indicator with bachelor’s degree as the reference category; and *cohort*, a dichotomous indicator describing if the child was from CANDLE (reference category) or BAMBAM to account for differences across cohorts unaccounted for by other covariates. In our study, minoritized race and ethnicity were largely colinear with cohort (see Table 1). Therefore, we did not adjust for race or ethnicity, nor did we interpret the estimated coefficients for the cohort variable as we cannot distinguish between cohort effects and those related to being in a minoritized group.

### 2.3 Analytic procedure

All analyses were conducted in R version 4.1.0 (R Core Team, 2020). To account for missing data on covariates, multiple imputation was conducted using the smcfcs package (Bartlett et al., 2022) in R with 100 imputations and 100 burn-in iterations per imputation. Trace plots were examined to ensure convergence.

We conducted separate linear regressions by outcome (i.e., reading and math) and computed a series of nested models to evaluate associations between instruction disruption group status and each outcome, with each subsequent model adjusting for additional covariates as follows: *Model A* included pre-pandemic vocabulary, months between vocabulary and the AAB, months between end of the Spring 2020 school year and AAB administration, and the cohort indicator; *Model B* added child-and family-level sociodemographic variables (child age, sex, and caregiver education) and child IEP status; and *Model C* added COVID-19 family hardships and caregiver pandemic-related traumatic stress. We compared nested models using Meng and Rubin (1992) D3 multiple imputation likelihood ratio test statistic and adjusted R^2^ values. We then conducted a series of effect moderation models (Models D1-D8) whereby an individual effect moderator was added to the fully adjusted model (Model C). Regression coefficients and standard errors were combined across imputations using Rubin’s pooling rules (Rubin, 1987).

## 3 Results

**FIGURE 3:**
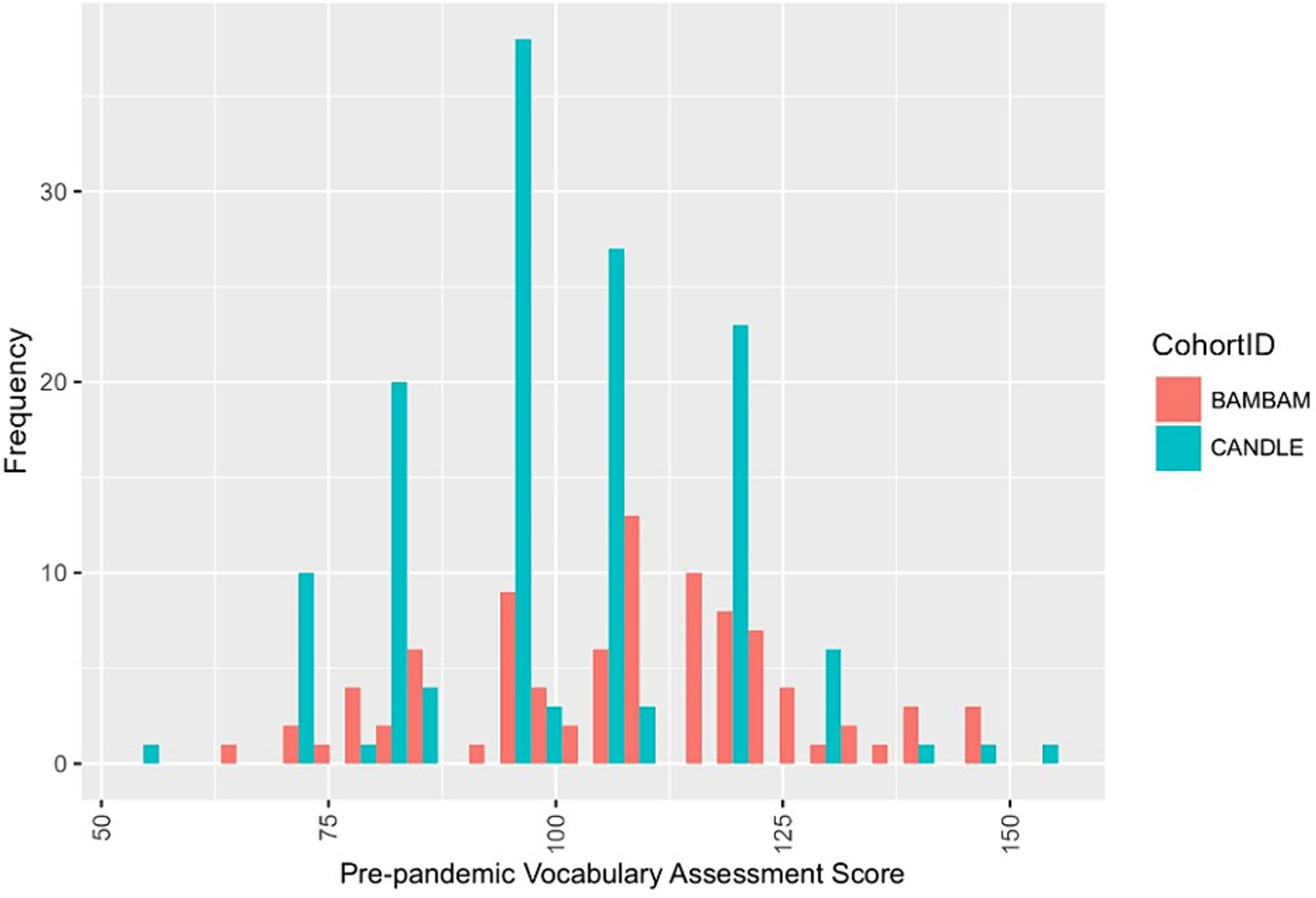
Pre-pandemic vocabulary score distribution by Cohort. Distribution of harmonized pre-pandemic vocabulary assessment score by ECHO cohort. Scores ranged from 53 to 153.

Participant characteristics are reported in Table 1. Children came from diverse racial, ethnic, and economic backgrounds: 43.6% were White, 36.2% Black, and 18.1% multiracial; 10.3% were Hispanic; and 53.9% had caregivers with less than a bachelor’s degree. On average, children were 9.9 years old (SD = 1.75) at the time of the AAB assessment. The sample was approximately evenly split between females (46.1%) and males (53.9%), and 18% had an IEP. Most caregivers (64.9%) reported at least one COVID-19-related family hardship, and on average, caregivers had moderate pandemic-related traumatic stress (M = 1.9, SD = 0.7). On average, children’s pre-pandemic vocabulary scores were 103.2 (SD = 17.9). During the pandemic, children on average scored 108.7 (SD = 19.7) on AAB reading and 92.8 (SD = 18.6) on AAB math, with a relatively normal distribution of scores within and across cohorts (Figure 3). Approximately a third of children in the sample (32.6%) experienced >4 weeks of instruction disruption with no learning in Spring 2020.

Linear regression results are provided in Table 2. Compared to our baseline Model A, the addition of child-and family-level demographics in Model B notably attenuated the association between instruction disruption and both outcomes. Further addition of family hardships and caregiver stress resulted in minimal additional attenuation. We only observed significant associations between instruction disruption duration and math achievement: children who experienced >4 weeks of disrupted learning scored 4.5 points [95% CI: −8.77, −0.22] lower on average compared to children whose experienced 4 weeks or less of learning disruption (Table 2, Model C: Math).

Several child-and family-level covariates were significantly associated with children’s AAB scores. Compared to children with lower pre-pandemic vocabulary scores, those with higher scores had slightly higher math ($\hat{b}=0.34$ [95% CI: 0.22, 0.46]) and reading ($\hat{b}=0.46$ [95% CI: 0.33, 0.59]) achievement during the pandemic. Being female was marginally significant for math, where females scored 3.42 points [95% CI: −0.36, 7.2] higher in math compared to males (*p* = 0.08). Children whose caregivers had less than a BA education scored 9.59 [95% CI: −14.2, −4.98] and 5.46 [95% CI: −10.4, −0.52] points lower on math and reading, respectively. Higher caregiver pandemic-related traumatic stress was marginally significant for math ($\hat{b}=2.71$ [95% CI: −5.74, 0.32], *p* = 0.08).

We found no evidence to suggest associations were moderated by child age, sex, IEP, pre-pandemic vocabulary, caregiver education, caregiver pandemic-related traumatic stress, or COVID-19-related family hardships (Supplementary Tables 1, 2).

## 4 Discussion

Children who experienced more than 4 weeks with no instruction (in-person or remote) had worse math performance compared to peers whose schools were closed for 4 weeks or less. The estimated 4.5-point difference in scores equates to nearly a one-third standard deviation lower score on average, which is three times higher than summer math learning loss estimates (Cooper et al., 1996) and larger than learning loss associated with student displacement from natural disasters (e.g., Sacerdote, 2012; Morrill and Westall, 2023). Reading scores were not associated with learning disruption duration, potentially due to reading being an activity that parents could easily accommodate without much guidance from educators, whereas math requires more technical knowledge. Even when parents engage in both reading and math activities at home, they spend twice as much time on reading compared to math (Napoli and Purpura, 2018), which may reflect higher levels of parent math anxiety (Maloney et al., 2015) that limited their ability to teach math when schools were closed for any type of learning – remote or otherwise. Our results reflect those found in group-level analyses (e.g., Hammerstein et al., 2021; König and Frey, 2022) and the broader phenomenon of stronger associations between math scores (compared to reading) and extended out-of-school time (e.g., Cooper et al., 1996; Kuhfled and Tarasawa, 2020). In the current study, analyses of individual-level data and staged modeling extends these findings by adjusting for confounding, enabling us to derive more valid estimates of the association between learning disruption and achievement. As most prior work on COVID-19 and student achievement uses group-level comparisons (e.g., school-level, historical cohorts), our results suggest prior findings may be over-estimates of the impact of pandemic-related school disruptions on child academic achievement. We further extend the literature by drawing on achievement data collected in-person and through the beginning of 2022 to examine the longer-term impact of Spring 2020 instruction disruptions, finding that students who completed the AAB farther from the start of the pandemic scored slightly worse in math compared to peers who completed the AAB closer to the start of the pandemic. Drawing on data through Spring 2021, Molnár and Hermann (2023) found 2nd–8th graders not only experienced an initial learning loss but a year later had nearly 0.25 of a standard deviation of accumulated learning loss compared to pre-pandemic peers. Our results further suggest COVID-19-related instruction disruptions may have even longer lingering impacts on student learning.

Individual level data can also be used to gain insights into factors beyond learning disruptions that may have impacted achievement. Our final models suggest that focusing on the length of disrupted learning fails to tell the entire story. Notably, children whose caregivers had less than bachelor’s education scored two-thirds of a standard deviation lower in math and one-third of a standard deviation lower in reading compared to children whose caregivers had a bachelor’s degree or higher. While the addition of pandemic-related factors only explained an additional 1–2% of the variance in our models, results showed caregiver stress was marginally associated with math scores, such that a one-point increase in stress was associated with approximately a fifth of a standard deviation lower math score. Caregivers who had lower educational attainment and experienced higher stress may have had more difficulty supplementing at-home math instruction due to increased psychological strain and limited resources and content knowledge to practice math directly with children. While this study was not explicitly designed to study these associations, our results indicate fruitful areas for future research that go beyond instruction disruption impacts.

Moreover, our individual-level data enabled investigation of the potential effect modification by important sociodemographic and pandemic factors. Though all tests were null, examining heterogeneity in the impact of the pandemic within different subgroups is of prime importance with respect to informing recovery efforts. Unfortunately, our study sample was largely underpowered for tests of effect modification. Mounting evidence suggests some children are more vulnerable to the effects of the pandemic (e.g., Hammerstein et al., 2021; Cohodes et al., 2022; König and Frey, 2022; Betthäuser et al., 2023), and larger studies with individual-level data like this one are necessary to examine differences.

### 4.1 Practical implications

The current study points to important considerations for elementary educators, schools, and families as the US continues to build back from the social and economic devastation of the COVID-19 pandemic. Even several years after the pandemic onset, students in our sample still showed lower math achievement regardless of how much time elapsed between school closures and AAB completion. Educators in schools that experienced extended learning disruptions in Spring 2020 may benefit from increased focus on math instruction, including incorporation of hands-on and materials-based math instruction that may have been missed during virtual instruction periods. High dosage math tutoring also holds promise, as Nickow et al. (2020) found students who engaged in at least 30 min of small group tutoring three to 5 days a week had 0.38 SD gains in math, nearly equivalent to the math learning loss found in this study.

Given the broader issue that math tends to be more impacted by extended out-of-school time, schools may benefit from building stronger foundational caregiver-school partnerships to support caregivers in at-home supplemental, hands-on math activities and instruction. Such partnerships require more than ensuring parents have the basic content knowledge necessary but also the confidence, as parental math anxiety can limit children’s math achievement (Maloney et al., 2015). Importantly, addressing broader family socioeconomic and psychosocial contextual factors is also critical, especially during highly stressful conditions such as the COVID-19 pandemic. Our study suggests that, in addition to learning disruptions resulting from school closures, child and family-level characteristics also played a role in student outcomes, unveiling important malleable mechanisms that can be targeted for future interventions. Wraparound services at the family level, such as caregiver mental health and material resource support, could have downstream impact on the child’s achievement and are therefore important factors for education policymakers to consider.

### 4.2 Limitations

This study is not without limitations. Our modest-sized sample from two primarily urban geographic locations may limit generalizability to children in other parts of the country. However, our findings are consistent with those of larger studies with more geographically diverse samples, and we uniquely contribute results drawing on individual-level data collected in person during a global pandemic—a feat unfeasible at large scale. Further, despite this being a partial cohort analysis, participants reflected the racial diversity of the full cohort samples but had caregivers who were, on average, more highly educated (Deoni et al., 2012; Sontag-Padilla et al., 2015). Such differences between our subsample and the full cohort may reflect a common challenge faced generally by other large pediatric cohort studies that continued data collection during the pandemic (e.g., Adolescent Brain Cognitive Development Study [ABCD]) whereby higher resourced families were able to participate and provide data during the COVID-19 pandemic (Yip et al., 2022). Thus, results presented here and those from other large-scale cohort studies conducted during the pandemic may not reflect the experiences of the hardest hit families who were not able to respond to surveys or assess children in person for academic achievement assessments mid-pandemic. Our findings may therefore underestimate the impact of COVID-19 learning disruptions and family contextual factors for these children.

Given children within each cohort came from the same geographical area, they were likely subject to district-level school closure plans, which would limit the variability of the number of weeks they experienced disrupted learning. Both Shelby County, TN and Providence, RI adopted various remote learning instructional strategies (e.g., digital and paper workbooks, recorded lessons) while school buildings were closed (Center on Reinviting Public Education, 2023) but known differences at the school and teacher level suggest learning disruptions did vary significantly (Hamilton and Ercikan, 2022). Therefore, while caregiver-report of instruction disruption may be slightly under or overestimated, caregivers were in the best position to know exactly how many weeks their children did not experience learning of any kind.

Like prior studies, we did not have individual-level pre-pandemic academic achievement data using the same during pandemic assessment (i.e., the AAB), which limited our ability to directly assess within-child changes resulting from the pandemic. Additionally, the skewed nature of our exposure measure required grouping children into those who experienced 4 weeks or less of no learning and those experienced more than 4 weeks of no learning. While this dichotomization was theoretically driven based on cumulative standard breaks that occur during a normal school year such as winter and spring vacation and thresholds for chronic absenteeism, the measure lacked granularity and may underestimate associations for those who experienced greater than 4 weeks of no learning. Indeed, 4 weeks into the pandemic, with only a third of US school districts offering a formal curriculum with instruction (Lake and Dusseault, 2020), many students likely experienced longer durations of no learning. Our measure of pre-pandemic vocabulary served as a robust proxy measure and enabled us to control for any pre-existing academic functioning differences. Finally, we did not have sufficient data on children’s 2020/21 school experiences, such as instructional type and quality, classroom environment, and curriculum content, which likely contribute to their achievement. However, prior research suggests the Spring 2020 school disruptions impacted achievement more so than later pandemic-related disruptions (König and Frey, 2022), suggesting Spring 2020 is the critical exposure period to assess as we do here.

Overall, this study extends our understanding of the impact of the COVID-19 pandemic on student achievement by providing one of the first investigations using individual-level data within a racially, ethnically, and economically diverse sample. Findings point to the potential need for remedial math instruction for children who experienced extended instruction disruptions and that children of caregivers with lower levels of education and higher stress may require the most support.

## Supplementary Material

supplement

## Figures and Tables

**TABLE 1:** Participant characteristics.

Variable	All Cohorts (*N* = 282)	CANDLE (*N* = 151)	BAMBAM (*N* = 131)
Age in years (M, SD) [range]	9.88 (1.75) [5.04, 12.85]	10.73 (0.56) [10.03, 11.99]	8.88 (2.1) [5.04, 12.85]
Female	130 (46.1%)	77 (51%)	53 (40.5%)
**Race**			
American Indian or Alaska Native	<5	<5	<5
Black	102 (36.2%)	93 (61.6%)	<10
Multiracial	51 (18.1%)	13 (8.6%)	38 (29%)
Other-identified Race	<5	<5	<5
White	123 (43.6%)	45 (29.8%)	78 (59.5%)
Missing	<5	<5	<5
Hispanic	29 (10.3%)	5 (3.3%)	24 (18.3%)
Missing	<5	<5	<5
IEP (1 = Yes)	51 (18.1%)	33 (21.9%)	18 (13.7%)
Missing	<5	<5	<5
**Caregiver education**			
< High school degree	11 (3.9%)	<10	<10
High school degree, GED or equivalent	25 (8.9%)	<20	<10
Some college, no degree/Associate’s degree/ Trade school	116 (41.1%)	89 (58.9%)	27 (20.6%)
Bachelor’s degree	49 (17.4%)	10 (6.6%)	39 (29.8%)
Masters degree, Professional or Doctorate Degree	74 (26.2%)	30 (19.9%)	44 (33.6%)
Missing	<10	<5	<10
**N COVID-19 family hardships**			
0 hardships	99 (35.1%)	63 (41.7%)	36 (27.5%)
1 hardship	80 (28.4%)	41 (27.2%)	39 (29.8%)
2 hardships	68 (24.1%)	32 (21.2%)	36 (27.5%)
3 hardships	15 (5.3%)	<10	<10
4 hardships	<10	<5	<5
5 hardships	<5	<5	<5
Missing	13 (4.6%)	<5	10 (7.6%)
Caregiver pandemic-related traumatic stress (M, SD)	1.9 (0.7)	1.8 (0.7)	2.2 (0.7)
Pre-pandemic vocabulary (M, SD) [range]	103.2 (17.9) [54,153]	99.8 (16.9) [54,153]	108.5 (18.3) [66,146]
Months vocabulary to AAB (M, SD) [range]	27.6 (7) [12, 65.6]	28.8 (4.4) [14.9, 38.1]	25.6 (9.5) [12, 65.5]
AAB Reading (M, SD) [range]	108.7 (19.7) [50,150]	106.1 (17.7) [58,150]	111.7 (21.5) [50,150]
AAB Math (M, SD) [range]	92.8 (18.6) [53,150]	89.2 (17.1) [53,147]	97 (19.5) [55,150]
Months end of Spring 2020 to AAB (M, SD) [range]	12.5 (3.5) [4.5,20.6]	13.1 (3.8) [6.1,23.6]	11.8 (3.1) [4.5,18.5]

AAB, Academic Achievement Battery; IEP, individualized education plan.

**TABLE 2:** Linear regression models predicting academic achievement from instruction disruption and covariates with mean centering.

Variable	Letter/word reading					Mathematical computation						
	Model A		Model B		Model C		Model A		Model B		Model C	
	$\hat{b}$	95%CI	$\hat{b}$	95%CI	$\hat{b}$	95%CI	$\hat{b}$	95%CI	$\hat{b}$	95%CI	$\hat{b}$	95%CI
Instruction disruption >4 weeks	−2.44	[−7.05, 2.17]	−1.19	[−5.8, 3.43]	−0.83	[−5.44, 3.77]	−5.76	[−10.1, −1.42]	−4.64	[−8.92, −0.36]	−4.5	[−8.77, −0.22]
Pre-pandemic vocabulary	0.5	[0.37, 0.63]	0.45	[0.32, 0.59]	0.46	[0.33, 0.59]	0.39	[0.27, 0.51]	0.33	[0.21, 0.45]	0.34	[0.22, 0.46]
Months vocabulary to AAB	0.3	[−0.03, 0.62]	0.16	[−0.23, 0.56]	0.19	[−0.2, 0.58]	0.18	[−0.11, 0.47]	0.24	[−0.1, 0.58]	0.25	[−0.09, −0.58]
Cohort	−1.95	[−6.62, 2.72]	−0.92	[−6.54, 4.7]	−2.07	[−7.78, 3.64]	−3.32	[−7.74, 1.09]	1.34	[−3.9, 6.59]	−0.15	[−5.18, 5.48]
Months end of Spring 2020 to AAB	−0.57	[−1.17, 0.02]	−0.55	[−1.14, 0.04]	−0.53	[−1.13, 0.06]	−0.69	[−1.25, −0.13]	−0.82	[−1.37, −0.27]	−0.77	[−1.32, −0.21]
Child Age			0.98	[−0.82, 2.78]	0.93	[−0.86, 2.71]			−0.76	[−2.37, 0.85]	−0.8	[−2.41, 0.8]
Female			0.42	[−3.63, 4.47]	0.84	[−3.21, 4.89]			3.11	[−0.68, 6.9]	3.42	[−0.36, 7.2]
IEP			−3.27	[−8.53, 1.99]	−3.54	[−8.78, 1.7]			2.75	[−2.15, 7.65]	2.55	[−2.33, 7.44]
Caregiver education < bachelor’s degree			−5.83	[−10.71, −0.95]	−5.46	[−10.4, −0.52]			−9.42	[−13.97, −4.86]	−9.59	[−14.2, −4.98]
COVID-19 hardships					−1.81	[−3.9, 0.28]					−0.7	[−2.61, 1.22]
Caregiver pandemic stress					−1.36	[−4.65, 1.93]					−2.71	[−5.74, 0.32]
(Intercept)	109.47	[105.96, 112.99]	102.58	[85.46, 119.7]	107.84	[89.54, 126.14]	95.56	[92.24, 98.87]	103.76	[88.48, 119.05]	110.76	[94.25, 127.27]
D3 statistic			*F*(4, 537840.27) = 2.05, p = 0.08, RIV = 0.09		*F*(2, 261742.32) = 2.62, *p* = 0.07, RIV = 0.1				*F*(4, 2311044.07) = 5.13, *p* < 0.001, RIV = 0.04		*F*(2, 569664.29) = 2.52, *p* = 0.08, RIV = 0.06	
Adjusted R^2^	0.28		0.29		0.3		0.25		0.29		0.3	

## Data Availability

The datasets presented in this study can be found in online repositories. The names of the repository/repositories and accession number (s) can be found at: individual-level de-identified ECHO data and the accompanying data dictionary are available in the NIH National Institute of Child Health and Human Development (NICHD) Data and Specimen Hub (DASH). To access these data, interested investigators can submit study proposals on the DASH website: https://dash.nichd.nih.gov.
