# Supplementary material for: The impact of COVID-19 school disruptions on children’s learning
Source: Front Educ (Lausanne). Author manuscript; Available in PMC 2024 Oct 8. (PMC11460541; doi:10.3389/feduc.2024.1295910)
Supplement: supplement [file NIHMS1993210-supplement-supplement.docx]

| **Supplementary Table 1**  *Letter/Word Reading Linear Regression Models with Effect Modification* | | | | | | | | | | | | | | | | |
| --- | --- | --- | --- | --- | --- | --- | --- | --- | --- | --- | --- | --- | --- | --- | --- | --- |
|  | **Model D1** | | **Model D2** | | **Model D3** | | **Model D4** | | **Model D5** | | **Model D6** | | **Model D7** | | **Model D8** | |
|  | *b̂* | 95%CI | *b̂* | 95%CI | *b̂* | 95%CI | *b̂* | 95%CI | *b̂* | 95%CI | *b̂* | 95%CI | *b̂* | 95%CI | *b̂* | 95%CI |
| School Closure >4 Weeks | -1.79 | [-9.81, 6.22] | -9.15 | [-36.69, 18.39] | -1.99 | [-7.93, 3.96] | -0.36 | [-5.44, 4.71] | 0.09 | [-8.14, 8.31] | 0.67 | [-5.67, 7.01] | -8.02 | [-20.56, 4.52] | -0.73 | [-5.37, 3.92] |
| Pre-pandemic vocabulary | 0.46 | [0.33, 0.6] | 0.46 | [0.33, 0.6] | 0.46 | [0.33, 0.6] | 0.46 | [0.33, 0.59] | 0.46 | [0.33, 0.6] | 0.46 | [0.32, 0.59] | 0.46 | [0.33, 0.59] | 0.43 | [0.27, 0.58] |
| Months vocabulary to AAB | 0.19 | [-0.2, 0.58] | 0.19 | [-0.2, 0.57] | 0.18 | [-0.2, 0.57] | 0.19 | [-0.2, 0.57] | 0.18 | [-0.21, 0.57] | 0.19 | [-0.2, 0.58] | 0.18 | [-0.21, 0.57] | 0.18 | [-0.21, 0.57] |
| Months end of Spring 2020 to AAB | -2.42 | [-1.12, 0.07] | -0.52 | [-1.12, 0.08] | -0.51 | [-1.11, 0.09] | -0.53 | [-1.13, 0.06] | -0.53 | [-1.13, 0.06] | -0.55 | [-1.15, 0.05] | -0.53 | [-1.12,  0.07] | -0.52 | [-1.12, 0.08] |
| Cohort | -0.53 | [-8.58, 3.74] | -2.3 | [-8.06, 3.47] | -2.17 | [-7.9, 3.55] | -2.09 | [-7.8, 3.63] | -2.12 | [-7.85, 3.61] | -1.9 | [-7.64, 3.85] | -2.19 | [-7.9, 3.52] | -2.23 | [-7.96, 3.49] |
| Child Age | 0.88 | [-0.91, 2.68] | 0.71 | [-1.15, 2.58] | 0.93 | [-0.85, 2.72] | 0.93 | [-0.85, 2.71] | 0.97 | [-0.85, 2.78] | 0.9 | [-0.89, 2.68] | 0.99 | [-0.8, 2.77] | 0.99 | [-0.8, 2.79] |
| Female | 0.8 | [-3.26, 4.86] | 0.83 | [-3.22, 4.87] | -0.02 | [-4.93, 4.89] | 0.93 | [-3.14, 5.01] | 0.87 | [-3.19, 4.92] | 0.81 | [-3.24, 4.86] | 0.93 | [-3.12, 4.97] | 0.92 | [-3.12, 4.97] |
| IEP | -3.57 | [-8.82, 1.69] | -3.53 | [-8.78, 1.71] | -3.72 | [-9.0, 1.57] | -2.67 | [-9.31, 3.96] | -3.53 | [-8.78, 1.72] | -3.57 | [-8.82, 1.67] | -3.59 | [-8.82, 1.65] | -3.44 | [-8.69, 1.82] |
| Caregiver education < bachelor’s degree | -5.45 | [-10.4,  -0.51] | -5.59 | [-10.56, -0.63] | -5.47 | [-10.41, -0.52] | -5.48 | [-10.42, -0.53] | -5.1 | [-10.65, 0.44] | -5.42 | [-10.36, -0.48] | -5.59 | [-10.53, -0.65] | -5.63 | [-10.58, -0.68] |
| COVID-19 hardships | -1.81 | [-3.9, 0.28] | -1.8 | [-3.89, 0.29] | -1.81 | [-3.9, 0.28] | -1.83 | [-3.92, 0.26] | -1.81 | [-3.9, 0.29] | -1.31 | [-3.89, 1.27] | -2.01 | [-4.12, 0.1] | -1.8 | [-3.89, 0.3] |
| Caregiver pandemic stress | -1.35 | [-4.65, 1.94] | -1.36 | [-4.65, 1.93] | -1.38 | [-4.67, 1.91] | -1.35 | [-4.64, 1.94] | -1.35 | [-4.64, 1.95] | -1.2 | [-4.52, 2.12] | -2.6 | [-6.47, 1.27] | -1.38 | [-4.67, 1.91] |
| School Closure* Cohort | 1.48 | [-8.1, 11.06] |  |  |  |  |  |  |  |  |  |  |  |  |  |  |
| School Closure*Age |  |  | 0.85 | [-1.88, 3.59] |  |  |  |  |  |  |  |  |  |  |  |  |
| School Closure*Sex |  |  |  |  | 2.7 | [-5.95, 11.35] |  |  |  |  |  |  |  |  |  |  |
| School Closure*IEP |  |  |  |  |  |  | -2.31 | [-13.14, 8.53] |  |  |  |  |  |  |  |  |
| School Closure* Caregiver education |  |  |  |  |  |  |  |  | -1.33 | [-11.0, 8.33] |  |  |  |  |  |  |
| School Closure* Hardships |  |  |  |  |  |  |  |  |  |  | -1.35 | [-5.33, 2.63] |  |  |  |  |
| School Closure* Caregiver stress |  |  |  |  |  |  |  |  |  |  |  |  | 3.84 | [-2.46, 10.14] |  |  |
| School Closure*Pre-pandemic vocabulary |  |  |  |  |  |  |  |  |  |  |  |  |  |  | 0.11 | [-0.16, 0.38] |
| (Intercept) | 108.42 | [89.82, 127.01] | 110.1 | [90.74, 129.46] | 108.28 | [89.01, 126.65] | 107.61 | [89.26, 125.96] | 107.23 | [88.35, 126.12] | 107.19 | [88.75, 125.63] | 109.98 | [91.3, 128.65] | 107.43 | [89.08, 125.78] |

Abbreviations: AAB, Academic Achievement Battery; IEP, individualized education plan; 95% CI: 95% Confidence Interval.

| **Supplementary Table 2**  *Mathematical Calculation Linear Regression Models with Effect Modification* | | | | | | | | | | | | | | | | |
| --- | --- | --- | --- | --- | --- | --- | --- | --- | --- | --- | --- | --- | --- | --- | --- | --- |
|  | **Model D1** | | **Model D2** | | **Model D3** | | **Model D4** | | **Model D5** | | **Model D6** | | **Model D7** | | **Model D8** | |
|  | *b̂* | 95%CI | *b̂* | 95%CI | *b̂* | 95%CI | *b̂* | 95%CI | *b̂* | 95%CI | *b̂* | 95%CI | *b̂* | 95%CI | *b̂* | 95%CI |
| School Closure >4 Weeks | -5.98 | [-13.29, 1.33] | 3.41 | [-21.54, 28.36] | -4.56 | [-10.09, 0.97] | -3.87 | [-8.61, 0.86] | -7.19 | [-14.8, 0.42] | -4.96 | [-10.85, 0.93] | -6.85 | [-18.43, 4.73] | -4.53 | [-8.81,  -0.24] |
| Pre-pandemic vocabulary | 0.34 | [0.22, 0.46] | 0.34 | [0.22, 0.46] | 0.34 | [0.22, 0.46] | 0.34 | [0.22, 0.46] | 0.34 | [0.22, 0.46] | 0.34 | [0.22, 0.46] | 0.34 | [0.22, 0.46] | 0.35 | [0.21, 0.49] |
| Months vocabulary to AAB | 0.25 | [-0.09, 0.58] | 0.25 | [-0.09, 0.58] | 0.25 | [-0.09, 0.58] | 0.25 | [-0.09, 0.58] | 0.26 | [-0.08, 0.59] | 0.25 | [-0.09, 0.58] | 0.24 | [-0.09, 0.58] | 0.25 | [-0.09, 0.59] |
| Months pandemic start to AAB | -0.76 | [-1.31,  -0.2] | -0.78 | [-1.34,  -0.22] | -0.77 | [-1.32,  -0.21] | -0.76 | [-1.32,  -0.21] | -0.77 | [-1.32,  -0.21] | -0.76 | [-1.32,  -0.2] | -0.76 | [-1.32,  -0.21] | -0.77 | [-1.33,  -0.21] |
| Cohort | -0.39 | [-6.11, 5.34] | 0.37 | [-5.0, 5.74] | 0.15 | [-5.2, 5.49] | 0.14 | [-5.2, 5.47] | 0.29 | [-5.05, 5.63] | 0.1 | [-5.26, 5.46] | 0.11 | [-5.23, 5.45] | 0.19 | [-5.16, 5.54] |
| Child Age | -0.87 | [-2.49, 0.75] | -0.61 | [-2.31, 1.09] | -0.81 | [-2.41, 0.8] | -0.8 | [-2.4, 0.81] | -0.93 | [-2.56, 0.7] | -0.8 | [-2.4, 0.81] | -0.78 | [-2.39, 0.82] | -0.82 | [-2.44, 0.79] |
| Female | 3.36 | [-0.44, 7.16] | 3.43 | [-0.36, 7.22] | 3.37 | [-1.22, 7.97] | 3.55 | [-0.27, 7.36] | 3.34 | [-0.45, 7.13] | 3.43 | [-0.36, 7.22] | 3.45 | [-0.35, 7.24] | 3.39 | [-0.4, 7.19] |
| IEP | 2.51 | [-2.39, 7.4] | 2.55 | [-2.34, 7.44] | 2.54 | [-2.38, 7.47] | 3.7 | [-2.48, 9.88] | 2.53 | [-2.36, 7.41] | 2.57 | [-2.32, 7.46] | 2.54 | [-2.35, 7.43] | 2.53 | [-2.36, 7.43] |
| Caregiver education < bachelor’s degree | -9.58 | [-14.2,  -4.96] | -9.43 | [-14.06, -4.8] | -9.59 | [-14.21, -4.97] | -9.61 | [-14.23, -5.0] | -10.61 | [-15.78, -5.44] | -9.59 | [-14.22, -4.97] | -9.63 | [-14.25, -5.01] | -9.54 | [-14.17, -4.91] |
| COVID-19 hardships | -0.7 | [-2.23, 1.22] | -0.72 | [-2.63, 1.2] | -0.7 | [-2.62, 1.22] | -0.72 | [-2.64, 1.2] | -0.72 | [-2.63, 1.2] | -0.85 | [-3.22, 1.51] | -0.76 | [-2.7, 1.18] | -0.7 | [-2.62 1.21] |
| Caregiver pandemic stress | -2.7 | [-5.74, 0.33] | -2.72 | [-5.75, 0.32] | -2.72 | [-5.75, 0.32] | -2.69 | [-5.73, 0.34] | -2.76 | [-5.79, 0.27] | -2.76 | [-5.83, 0.3] | -3.12 | [-6.69, 0.45] | -2.71 | [-5.74, 0.33] |
| School Closure* Cohort | 2.28 | [-6.55, 11.12] |  |  |  |  |  |  |  |  |  |  |  |  |  |  |
| School Closure*Age |  |  | -0.81 | [-3.31, 1.73] |  |  |  |  |  |  |  |  |  |  |  |  |
| School Closure*Sex |  |  |  |  | 0.14 | [-7.97, 8.26] |  |  |  |  |  |  |  |  |  |  |
| School Closure*IEP |  |  |  |  |  |  | -3.06 | [-13.15, 7.02] |  |  |  |  |  |  |  |  |
| School Closure* Caregiver education |  |  |  |  |  |  |  |  | 3.9 | [-5.11, 12.9] |  |  |  |  |  |  |
| School Closure* Hardships |  |  |  |  |  |  |  |  |  |  | 0.41 | [-3.26, 4.08] |  |  |  |  |
| School Closure* Caregiver stress |  |  |  |  |  |  |  |  |  |  |  |  | 1.26 | [-4.53, 7.05] |  |  |
| School Closure*Pre-pandemic vocabulary |  |  |  |  |  |  |  |  |  |  |  |  |  |  | -0.03 | [-0.27, 0.22] |
| (Intercept) | 111.65 | [94.81, 128.5] | 108.7 | [91.06, 126.34] | 110.8 | [94.2, 127.39] | 110.47 | [93.91, 127.03] | 112.57 | [95.51, 129.64] | 110.97 | [94.32, 127.62] | 111.46 | [94.6, 128.33] | 110.85 | [94.28, 127.43] |

Abbreviations: AAB, Academic Achievement Battery; IEP, individualized education plan; 95% CI: 95% Confidence Interval
